# Preferences of Pregnant Women and Healthcare Professionals on First‐Trimester Ultrasound Screening for Fetal Anomalies: A Discrete Choice Experiment

**DOI:** 10.1002/pd.70115

**Published:** 2026-03-03

**Authors:** Eline E. R. Lust, Kim Bronsgeest, Ian Smith, Lidewij Henneman, Neeltje M. T. H. Crombag, Caterina M. Bilardo, Robert‐Jan H. Galjaard, Esther Sikkel, Mireille N. Bekker, Monique C. Haak

**Affiliations:** ^1^ Department of Obstetrics and Gynecology University Medical Center Utrecht Utrecht the Netherlands; ^2^ Department of Obstetrics and Gynecology Leiden University Medical Center Leiden the Netherlands; ^3^ Centre for Health Protection National Institute of Public Health and the Environment Bilthoven the Netherlands; ^4^ Department of Human Genetics and Amsterdam Reproduction and Development Research Institute Amsterdam UMC Location Vrije Universiteit Amsterdam Amsterdam the Netherlands; ^5^ Department of Obstetrics and Gynecology Amsterdam the Netherlands; ^6^ Department of Clinical Genetics Erasmus University Medical Center Rotterdam the Netherlands; ^7^ Department of Obstetrics and Gynecology Radboud University Medical Center Nijmegen the Netherlands

**Keywords:** abnormalities, attitude of health personnel, choice behavior, discrete choice experiment, early diagnosis, fetal screening, fetus, patient preference, prenatal ultrasonography, surveys and questionnaires

## Abstract

**Objective:**

The first‐trimester anomaly scan (FTAS) allows early detection of fetal structural anomalies. Decisions on timing and scan protocol influence its performance. Understanding how pregnant women and healthcare professionals value these aspects is essential for shaping screening programs. This study aims to identify which aspects of first‐trimester ultrasound screening are considered most important.

**Method:**

A discrete choice experiment was conducted between July and August 2025. It included five attributes: sensitivity (30%, 50%, 90%), gestational age at diagnosis (12, 16, 22 weeks), false positive rate (20%, 40%, 60%), false negative rate (0.1%, 1%, 5%) and types of anomalies detected (most severe only vs. most severe and other). Additionally, general attitudes toward the FTAS were assessed.

**Results:**

Questionnaires were completed by 1963 pregnant women who opted for FTAS and 1068 healthcare professionals (203 medical doctors, 865 primary care professionals). Most important attributes for pregnant women and healthcare professionals were sensitivity and false positive rate. 85.1% of pregnant women and 58.1% of healthcare professionals (strongly) agreed that the FTAS should be available to pregnant women.

**Conclusion:**

Pregnant women and healthcare professionals support FTAS availability. Optimization should focus on maximizing sensitivity and minimizing false positive rates, whereas gestational age at diagnosis and the range of anomalies detected are considered less important in influencing preferences.

## Summary

- What's already known about this topic?
  ◦ The first‐trimester anomaly scan (FTAS) detects fetal structural anomalies earlier in pregnancy
  ◦ Preferences of pregnant women and healthcare professionals regarding different aspects of first‐trimester ultrasound screening are unclear, yet essential for optimal FTAS implementation.
- What does this study add?
  ◦ Pregnant women who opt for FTAS and healthcare professionals both prioritize high sensitivity and low false positive rates.
  ◦ Most pregnant women in this study (85.1%) and a majority of healthcare professionals (58.1%) support FTAS availability for pregnant women.

## 1 Introduction

First‐trimester ultrasound screening for fetal anomalies is offered across many developed countries [1]. In the Netherlands, a first‐trimester anomaly scan (FTAS) has been offered since September 2021 in the nationwide prenatal screening program as part of an implementation study, the “Implementation of First Trimester Anomaly Scan” (IMITAS) study [2]. The aim was to enable early detection of major structural anomalies, thereby supporting autonomous reproductive decision‐making. FTAS was successful in identifying anomalies such as anencephaly and abdominal wall defects with detection rates approaching 100% [2, 3]. Many other anomalies, such as severe heart defects, were also found via the FTAS, though with lower detection rates. For example, a detection rate of 59.8% was described for heart defects with a severely asymmetrical four‐chamber view, such as hypoplastic left heart syndrome [3]. Such anomalies represented a substantial portion of the diagnostic yield of the FTAS [2, 3].

Despite the promise of the FTAS, the diversity of detectable anomalies, each associated with different detection rates, and the influence of gestational age (GA) on sonographic performance mean that the ultrasound screening offer can be adjusted, for example, by varying the GA at which the scan is offered. Offering the FTAS at an early GA focuses on detection of major and lethal anomalies but increases the likelihood of not recognizing other clinically relevant anomalies. In contrast, offering the scan at a later GA allows a more extensive protocol and broader anomaly detection, although this may result in longer diagnostic trajectories and higher false‐positive rates. These factors complicate the trade‐off between potential benefits and harm, such as earlier diagnosis with more time for reproductive decision‐making and anxiety caused by false‐positive results [2, 4]. It is unknown how pregnant women and healthcare professionals involved in prenatal screening value the different aspects of first‐trimester ultrasound screening. Identifying their preferences is essential for tailoring FTAS implementation effectively. Therefore, the primary aim of this study was to assess and compare the aspects of first‐trimester ultrasound screening that are most important to pregnant women and healthcare professionals, in order to guide future FTAS implementation.

## 2 Method

### 2.1 Study Design

A cross‐sectional study was conducted using an online questionnaire. Approval for the study was granted by the Dutch Ministry of Health, Welfare, and Sport (license 3219987‐1012059‐PG), based on positive advice by the Health Council of the Netherlands (2021/30). The present study was a predefined component of the ‘Implementation of First Trimester Anomaly Scan’ (IMITAS) study and was included in the original grant proposal and ethical approval. Informed consent was incorporated in the online questionnaire, which included study information and contact details for questions.

### 2.2 Setting

This study was conducted within the IMITAS study, a nationwide study evaluating FTAS implementation within the Dutch prenatal screening program [2]. The national prenatal screening program further comprises non‐invasive prenatal testing (NIPT) and the second‐trimester anomaly scan (SAS), which is performed at GA 18–21 weeks [2, 5] Women could undergo either NIPT first or FTAS first, as there was no predetermined order for conducting the tests.

### 2.3 Study Population

This study was conducted between July and August 2025. It included pregnant women and healthcare professionals involved in prenatal screening across the Netherlands. Pregnant women were eligible for inclusion if they were 16 years or older, proficient in Dutch and had opted for the FTAS after prenatal counseling. Healthcare professionals eligible for inclusion were prenatal counselors, sonographers, midwives, fetal medicine specialists, obstetricians, pediatric specialists (e.g., pediatric cardiologists) and clinical geneticists. Responses were collected anonymously. Pregnant women who opted for FTAS were identified and recruited by the national prenatal registration database (Peridos). Recruitment of healthcare professionals was conducted through direct outreach to midwifery practices and hospitals, as well as via social media and mass emails distributed through professional associations.

### 2.4 Survey

The survey collected general demographic information, followed by a discrete choice experiment (DCE) designed to explore how respondents value different aspects of first‐trimester ultrasound screening. Subsequently, respondents answered questions regarding their attitudes toward the FTAS and additionally for professionals' questions about the preferred sequencing of NIPT relative to the FTAS (Supplementary material (Supplemental S1)).

### 2.5 DCE Design

The DCE design was developed in line with established best practice guidelines [6]. A DCE is commonly used in healthcare settings to quantify the preferences of patients or healthcare professionals [7]. In a DCE, respondents are given a series of choice tasks where they select their preferred option from two or more alternative treatment profiles. These alternatives depict treatments through a series of characteristics, known as attributes, each with varying levels that reflect realistic values. After DCE completion, econometric models are used to generate estimates for each attribute, from which the relative importance of these attributes can be determined [8].

Potential attributes for the DCE were derived from scientific literature and from the previously conducted IMITAS studies [2, 3, 9, 10]. Most relevant attributes were selected by members of the research team (EL, KB, MH, MB, IS). The selected attributes (sensitivity, GA at diagnosis, false positive rate, false negative rate, types of anomalies detected) are provided in Table 1 together with their levels and explanations. An example of a choice task is shown in Supplementary material (Supplemental S2).

**TABLE 1:** Attributes and attribute levels included in the discrete choice experiment.

Attributes	Levels	Explanation
Sensitivity	30% 50% 90%	The chance that the ultrasound detects the anomaly in cases where the fetus does have an anomaly.
Gestational age at diagnosis	At 12 weeks of pregnancy At 16 weeks of pregnancy At 22 weeks of pregnancy	The moment when the diagnosis becomes clear, after follow‐up testing has been performed. It is clear what this means for the prognosis.
False positive rate	20% 40% 60%	The chance that the ultrasound (incorrectly) shows something abnormal, while in reality the fetus is unaffected.
False negative rate	1 in 1000 (0.1%) 1 in 100 (1%) 1 in 20 (5%)	The chance that anomalies in the fetus are missed during the ultrasound.
Types of anomalies detected	Only the most severe anomalies Most severe and also other anomalies	Whether the ultrasound detects only the most severe anomalies. These are anomalies that are not compatible with life or severely limit it, such as an open skull or spina bifida. Or whether the ultrasound also detect other anomalies. The impact of these on quality of life can vary. Examples include a heart defect or clubfoot. All of the above‐mentioned anomalies can also be detected at the second‐trimester anomaly scan.

### 2.6 Statistical Analysis

Only participants who fully completed both the DCE and the attitude questions were included in the analysis. Descriptive statistics using *R* Version 4.3.1 (The *R* Foundation for Statistical Computing) were used to summarize baseline characteristics and study‐specific questions. Differences in attitude between pregnant women and healthcare professionals were assessed using Pearson's χ^2^ test and statistical significance was set at *p* < 0.05.

A D‐efficient main‐effects design was generated using the online version of Ngene, provided through the SurveyEngine platform. The *β*‐prior estimates were derived within the research team by rating the importance of each attribute relative to the most important one. This resulted in a single block of 12 choice sets with two alternatives and an one opt‐out option to which participants were randomized. The opt‐out option was labeled ‘none of the above’, allowing respondents to indicate that they did not prefer either of the two alternatives. The survey and DCE were pretested in a small sample of pregnant women and healthcare professionals. Sample size was calculated using the rule of thumb as proposed by Johnson and Orme et al. [11, 12, 13], resulting in a minimum of 42 respondents for each group. Healthcare professionals were classified as medical doctors (including maternal‐fetal medicine physicians, OB/GYNs, pediatric specialists and clinical geneticists) or as primary care professionals (including prenatal counselors, sonographers, midwives). The DCE data were analyzed using a multinomial logit model. Effects coding was used [14]. An alternative‐specific constant was included in the model to account for possible left‐right bias and a constant was incorporated for the opt‐out alternative. The relative importance of each attribute was calculated by dividing the *β*‐coefficient of each attribute by the highest *β*‐coefficient [15]. Statistical significance was set at *p* < 0.05. Analyses were performed using the Apollo software package in *R* Version 4.3.1.

## 3 Results

A total of 6228 questionnaires were started, including 3739 by pregnant women and 2545 by healthcare professionals. Of these, 3197 were excluded due to non‐completion of both the DCE and attitude questionnaire (1739 pregnant women, 1458 healthcare professionals), with most non‐completers discontinuing after the introduction of the questionnaire. An additional 56 respondents were excluded, including eight healthcare professionals and 37 pregnant women who did not provide informed consent, two pregnant women who were younger than 16 years, and nine pregnant women who had not opted for the FTAS. The final sample comprised 3031 respondents, including 1963 pregnant women and 1068 healthcare professionals involved in prenatal screening. The response rate among pregnant women was 37.7% (2011/5331). For healthcare professionals, the response rate was unknown, as recruitment occurred via multiple methods including social media. Baseline characteristics of the pregnant women and healthcare professionals are presented in Tables 2 and 3, respectively. Most (69.2%, *n* = 1359) pregnant women had a high level of education and 11.6% (*n* = 227) already had the FTAS during the current pregnancy. Of the healthcare professionals, 203 (19.0%) were medical doctors (MDs) and 865 (81.0%) were primary care professionals (PCPs). Subsequent sections describe the results of the DCE and the attitude questionnaire.

**TABLE 2:** Baseline characteristics of pregnant women (*n* = 1963).

Characteristics	Values
Gestational age, weeks + days (median, IQR)	11 + 5 (10+5‐12+5)
Already had FTAS during current pregnancy, *n* (%)	227 (11.6)
Primigravida, *n* (%)	
Yes	941 (47.9)
No	1022 (52.1)
*Had FTAS during previous pregnancy, n* (%)	*646/1022 (63.2)*
Maternal age, years (mean ± SD)	31.6 ± 4.3
Education level, *n* (%)^a^ (missing 21)	
Low	74 (3.8)
Intermediate	497 (25.6)
High	1359 (69.2)
Other	12 (0.6)
Ethnicity (missing 17)	
Dutch	1646 (84.6)
Other western	123 (6.3)
Non‐western	151 (7.8)
Other	26 (1.3)
Religious, *n* (%) (missing 56)	
Yes	382 (20.0)
No	1517 (79.5)
Other	8 (0.4)

Abbreviations: *FTAS*, first‐trimester anomaly scan; IQR, interquartile range; SD, standard deviation.

^a^ Education level: Level categorized as low (no education or highest attained educational level primary school, low level of secondary school, lower vocational training), intermediate (middle vocational training or high level of secondary school), or high (higher vocational training or university/doctorate).

**TABLE 3:** Baseline characteristics of healthcare professionals (*n* = 1068).

Characteristics	Values
Profession, *n* (%)	
Medical doctors	203 (19.0)
*Subcategories (% within MDs)*	
*OB/GYN*	*145 (71.4)*
*Pediatric specialist* ^a^	*29 (14.3)*
*Clinical geneticist*	*16 (7.9)*
*Physicians performing diagnostic scans in referral center*	*13 (6.4)*
Primary care professionals	865 (81.0)
*Subcategories (% within PCPs)* *^b^*	
*Prenatal counselor*	569 (65.8)
*Sonographer*	523 (60.5)
*Midwife*	702 (81.2)
Setting, *n*(%)^c^	
Primary care	762 (71.3)
Secondary care	242 (22.7)
Tertiary care	155 (14.5)
Years in profession, *n* (%)	
< 5	178 (16.7)
5–15	366 (34.3)
> 15	524 (49.1)

Abbreviations: *MDs*, medical doctors; *PCPs,* primary care professionals.

^a^ Pediatric specialist: Including pediatric cardiologist (*n =* 12), pediatric urologist (*n =* 3), pediatric nephrologist (*n =* 3), pediatric neurologist (*n =* 1), pediatric surgeon (*n =* 4), other (*n =* 6).

^b^ Subcategories: Multiple answers possible; percentages are calculated based on total *n =* 865; respondents with multiple roles were counted in each relevant category.

^c^ Setting: Multiple answers possible; percentages are calculated based on total *n =* 1068.

### 3.1 DCE

**FIGURE 1:**
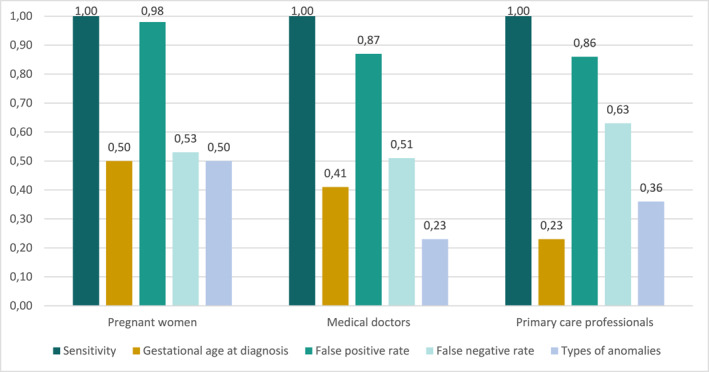
Relative importance of attributes for pregnant women, medical doctors and primary care professionals. This figure illustrates the importance of each attribute relative to the most important attribute, set at 1.00, which was sensitivity for all groups.

Table 4 shows the results of the DCE analysis. All attributes were found to be significant for decision‐making among all groups for at least one of their levels. Preferences for first‐trimester ultrasound screening followed a logical pattern and increased with higher sensitivity, earlier GA at diagnosis, fewer false positives, lower percentage of false negatives and the detection of both most severe and other anomalies. No left‐right bias was found. The relative attribute importance is presented in Figure 1. For all three groups, sensitivity was the most important attribute followed by false positive rate and thereafter false negative rate. The difference between sensitivity and false positive rate was nominal for pregnant women, while sensitivity was 1.15 and 1.16 times more important than false positive rate for MDs and PCPs, respectively. For pregnant women, gestational age at diagnosis and type of anomalies were the least important attributes, although they were only half as important as the most important attribute when selecting an option. For MDs, the least important attribute was the type of anomalies, whereas for PCPs, it was gestational age at diagnosis. Compared with these attributes, the level of sensitivity was 4.3 times more important when making a choice.

**TABLE 4:** Preferences of pregnant women, medical doctors and primary care professionals on first‐trimester ultrasound screening for fetal anomalies.

Attribute levels	Pregnant women (*n* = 1963) β (SE)	*P*	Medical doctors (*n* = 203) β (SE)	*p*	Primary care professionals (*n* = 865) β (SE)	*p*
*Sensitivity*						
30% (ref.)	−0.553 (0.020)		−1.002 (0.082)		−0.835 (0.036)	
50%	0.039 (0.024)	0.107	0.120 (0.073)	0.101	−0.020 (0.039)	0.621
90%	0.514 (0.017)	< 0.001	0.881 (0.059)	< 0.001	0.855 (0.032)	< 0.001
*GA at diagnosis*						
12 weeks (ref.)	0.302 (0.014)		0.335 (0.057)		0.215 (0.026)	
16 weeks	−0.072 (0.021)	< 0.001	0.098 (0.060)	0.105	−0.048 (0.033)	0.142
22 weeks	−0.230 (0.018)	< 0.001	−0.433 (0.068)	< 0.001	−0.166 (0.032)	< 0.001
*False positive rate*						
20% (ref.)	0.379 (0.021)		0.758 (0.067)		0.591 (0.029)	
40%	0.283 (0.030)	< 0.001	0.122 (0.072)	0.092	0.263 (0.036)	< 0.001
60%	−0.662(0.021)	< 0.001	−0.880 (0.071)	< 0.001	−0.854 (0.033)	< 0.001
*False negative rate*						
0.1% (ref.)	0.269 (0.016)		0.386 (0.052)		0.381 (0.026)	
1%	0.023 (0.029)	0.420	0.196 (0.086)	0.023	0.305 (0.041)	< 0.001
5%	−0.292 (0.021)	< 0.001	−0.583 (0.072)	< 0.001	−0.685 (0.037)	< 0.001
*Types of anomalies detected*						
Only most severe (ref.)	−0.268 (0.011)		−0.219 (0.037)		−0.304 (0.019)	
Most severe and other	0.268 (0.010)	< 0.001	0.219 (0.037)	< 0.001	0.304 (0.019)	< 0.001
*ASC choice 1*	0.015 (0.018)	0.417	0.078 (0.059)	0.190	0.018 (0.029)	0.537
*ASC opt‐out*	−0.735 (0.041)	< 0.001	0.081 (0.124)	0.512	−0.143 (0.056)	0.011

Abbreviations: *ASC*, alternative specific constant; *GA*, gestational age; *Ref*, reference category; SE, standard error.

*Note:* Positive *β*‐coefficients indicate increased utility, while negative *β*‐coefficients indicate decreased utility. An alternative specific constant (ASC choice 1) was included to correct for possible left‐right bias and a constant was incorporated for the opt‐out alternative (ASC opt‐out).

A key difference between the groups was in their valuation of the opt‐out. Pregnant women showed a clear preference for an FTAS compared with opting out (*p* < 0.001), while for both MDs and PCPs, this preference was dependent on the characteristics of the FTAS. The results indicate that MDs and PCPs would likely only accept an FTAS if it met certain criteria (such as a minimum sensitivity of 50%, a maximum false positive rate of 40%, a maximum false negative rate of 1%, a diagnosis occurring around or before 16 weeks, and screening for both the most severe and other conditions). It should also be noted that while pregnant women generally would opt for an FTAS, they would opt out if multiple attributes were at their worst level. Additionally, both MDs and PCPs showed a greater range in the coefficients for the test accuracy attributes, indicating that the changes in these attributes had a greater impact on their choices.

Regarding improving the FTAS, for pregnant women the greatest gains could be found in improving the false positive rate from 60% to 40%, followed by improving the sensitivity from 30% to 50%, testing for most severe and other anomalies, improving the sensitivity from 50% to 90%, moving the gestational age at diagnosis from 16 to 12 weeks, and improving the false negative rate from 5% to 1%.

For MDs, the greatest gains could be found in improving the sensitivity from 30% to 50%, followed by improving the false positive rate from 60% to 40%, improving the false negative rate from 5% to 1%, improving the sensitivity from 50% to 90%, improving the false positive rate from 40% to 20%, moving the gestational age at diagnosis from 16 to 12 weeks, and testing for most severe and other anomalies.

For PCPs, the greatest gains could be found in improving the false positive rate from 60% to 40%, followed by improving the false negative rate from 5% to 1%, improving the sensitivity from 50% to 90%, improving the sensitivity from 30% to 50%, and testing for most severe and other anomalies.

Pregnant women perceived the shift in the timing of diagnosis from 12 to 16 weeks of gestation as negative to their preferences, whereas for healthcare professionals it was not important for their preferences. While there is a consistent negative association between preferences and rate of false positives, the findings show that both pregnant women and primary care providers would accept a worsening of false positive rates (from 20% to 40%) if it meant a broader screening. Medical doctors would not accept that.

### 3.2 Attitude Questionnaire

Table 5 presents the attitudes of pregnant women and healthcare professionals toward the FTAS. Responses regarding whether the FTAS should be available differed significantly between pregnant women and the total group of healthcare professionals (*p* < 0.001), with pregnant women being more positive compared to health professionals. Overall, 85.1% (1671/1963) of the pregnant women and 58.1% (621/1068) of the healthcare professionals (strongly) agreed that the FTAS should be available to pregnant women. Opinions on the timing of NIPT relative to the FTAS varied, as 42.5% (454/1068) of healthcare professionals favored performing NIPT before the FTAS and 45.5% (486/1068) supported allowing pregnant women to choose the order. In total, 29.9% (587/1963) of pregnant women (strongly) agreed that the questionnaire was difficult to complete, compared with 36.3% (388/1068) of the healthcare professionals.

**TABLE 5:** General attitudes of pregnant women and healthcare professionals toward the FTAS.

Statement or question	Pregnant women (*n* = 1963), %	All healthcare professionals (*n* = 1068), %	Medical doctors (*n* = 203), %	Primary care professionals (*n* = 865), %
The FTAS should be available to pregnant women				
Strongly disagree	84 (4.3)	68 (6.4)	16 (7.9)	52 (6.0)
Disagree	43 (2.2)	140 (13.1)	20 (9.9)	120 (13.9)
Neutral	165 (8.4)	239 (22.4)	46 (22.7)	193 (22.3)
Agree	564 (28.7)	332 (31.1)	58 (28.6)	274 (31.7)
Strongly agree	1107 (56.4)	289 (27.1)	63 (31.0)	226 (26.1)
The FTAS should be permanently implemented into prenatal care				
Yes, exactly as it is currently offered	—	420 (39.3)	74 (36.5)	346 (40.0)
Yes, but with modifications to the current offering	—	306 (28.7)	57 (28.1)	249 (28.8)
No, it should not be implemented	—	148 (13.9)	32 (15.8)	116 (13.4)
I don't know	—	194 (18.2)	40 (19.7)	154 (17.8)
What is your opinion on the timing of the NIPT in relation to the FTAS?				
The NIPT should take place before the FTAS	—	454 (42.5)	117 (57.6)	337 (39.0)
The FTAS should take place before the NIPT	—	27 (2.5)	13 (6.4)	14 (1.6)
Pregnant women should decide which test they undergo first	—	486 (45.5)	45 (22.2)	441 (51.0)
I don't know	—	57 (5.3)	11 (5.4)	40 (4.6)
Other	—	44 (4.1)	11 (5.4)	33 (3.8)
If the NIPT is performed, should its result be awaited before performing the FTAS?				
Yes	—	231 (21.6)	91 (44.8)	140 (16.2)
No	—	682 (63.9)	75 (36.9)	607 (70.2)
I don't know	—	96 (9.0)	25 (12.3)	71 (8.2)
Other	—	59 (5.5)	12 (5.9)	47 (5.4)

Abbreviations: *FTAS*, first‐trimester anomaly scan; *NIPT*, non‐invasive prenatal testing.

## 4 Discussion

### 4.1 Main Findings

Our DCE findings indicate that, while all groups prefer first‐trimester ultrasound screening that offers both high accuracy and an earlier GA at diagnosis, respondents would be willing to accept a later GA at diagnosis if this achieves a higher sensitivity. Although both sensitivity and false positive rates were considered important, pregnant women viewed these attributes as similarly important, whereas healthcare professionals clearly prioritized sensitivity over false positive rates. Most pregnant women who opted for FTAS and a majority of healthcare professionals involved in prenatal screening (strongly) agreed that the FTAS should be available to pregnant women.

In our study, pregnant women placed a high value on high sensitivity for detecting fetal anomalies. This aligns with findings from an earlier DCE on third‐trimester ultrasound, where detection of abnormal fetal growth was highly valued, suggesting that pregnant women generally prioritize accurate information about fetal health [16]. Previous research has found that pregnant women value prenatal scans, such as the second‐trimester anomaly scan, as a way to be reassured about the health of the child [17]. Healthcare professionals in our study similarly prioritized high sensitivity.

An international DCE on preferences for non‐invasive and invasive prenatal testing for Down syndrome reported that women placed relatively greater emphasis on test safety and comprehensive information, while healthcare professionals prioritized test accuracy and earlier timing of testing [18]. This difference compared to our study may be attributable to the distinct context of screening by ultrasound, which does not involve a direct procedure‐related miscarriage risk and methodological differences, including the selection of attributes included in the DCEs.

Secondly, the attribute false positive rate was highly valued by all groups. This may reflect concerns about the psychological impact of uncertain or initially abnormal results after first‐trimester ultrasound screening. Previous studies have shown that such findings can elevate anxiety during pregnancy and, in some cases, lead to long‐term adverse consequences [4, 19, 20, 21]. This is supported by the somewhat lower valuation of the false negative rate in our study, suggesting that respondents may prefer to accept the risk of missing an anomaly rather than face unnecessary worry, have unnecessary diagnostic testing, or make reproductive decisions based on uncertain findings. Together, the prioritization of sensitivity and false positive rate highlights the trade‐offs involved in first‐trimester ultrasound screening. While the FTAS within the Dutch prenatal screening program shows a high sensitivity for first‐trimester major congenital anomalies, its sensitivity for ‘all‐anomalies‐combined’ is lower [2]. Expanding the scope of detection, through a more extensive protocol, is likely to increase the false‐positive rate. The execution of a detailed protocol at a national level may yield different results compared with single‐center studies conducted in specialized secondary or tertiary centers. This aspect remains important, as false positive findings can cause unnecessary distress for parents, increase healthcare costs and have resource implications. However, some false positives are inevitable, as a low referral threshold is intentionally maintained to enhance detection and to ensure access to specialist support for sonographers working in primary care settings. In our study, one of the greatest gains for all respondents was the reduction in the false positive rate. Therefore, future research should assess the false positive rate and explore the specific parameters that contribute to false positive findings in first‐trimester ultrasound screening.

Previous studies suggest that both parents and healthcare professionals generally prefer to identify serious anomalies as early as possible [22, 23, 24]. In our study, however, we encountered differences between the groups. Pregnant women generally preferred to undergo first‐trimester ultrasound screening, regardless of the specific test characteristics, whereas PCPs required higher criteria and MDs had the strictest criteria for accepting the test. In line with this, pregnant women placed relatively more importance on the GA at diagnosis and types of anomalies that are detected compared with healthcare professionals. This may reflect that earlier detection allows parents with more time for additional diagnostic testing and reproductive decision‐making, and that, if fetal anomalies are present, parents prefer to be informed early in pregnancy [2, 25]. Supporting this, other studies have reported that most pregnant women wish to be informed as early as possible in pregnancy about fetal anomalies [23, 24]. For some parents, earlier diagnosis of severe fetal anomalies may also allow for earlier termination of pregnancy, which is associated with a lower psychological impact compared to late pregnancy termination [10, 26, 27, 28]. In contrast, MDs and PCPs placed relatively less importance on the timing of diagnosis and types of anomalies. This may reflect their focus on the overall accuracy of the ultrasound and on what is technically feasible in practice, whereas pregnant women may consider these factors more hypothetically. Additionally, the preference of healthcare professionals for more accurate tests may be driven by the need for certainty in decision‐making and their awareness of the potential consequences of unnecessary diagnostic testing. Pregnant women may underestimate their personal risk of experiencing adverse outcomes. This interpretation is supported by findings from an interview study which showed that information about abnormal ultrasound findings often came unexpectedly to most parents [29].

In our study, most (85.1%) pregnant women and a majority (58.1%) of healthcare professionals (strongly) agreed that the FTAS should be available to pregnant women, while only 6.5% of pregnant women and 19.5% of healthcare professionals (strongly) disagreed. Rates were similar between MDs (59.6%) and PCPs (57.9%). The higher proportion of pregnant women compared to healthcare professionals may reflect a generally positive attitude toward prenatal ultrasound, often perceived as reassuring and emotionally meaningful, enhancing maternal‐fetal attachment [30]. However, pregnant women in our study had opted for the FTAS, indicating a potential selection bias. However, many pregnant women in our study did not yet undergo the FTAS or had experienced normal results, which may have shaped their attitudes more by expectations than by personal experience with abnormal findings. In contrast, healthcare professionals are more familiar with both the benefits and potential harms of FTAS screening. Nevertheless, 39.3% (420/1068) of healthcare professionals stated that the FTAS should be implemented as it is currently offered and 28.7% (306/1068) with modifications, together representing a majority. Among MDs, 57.6% favored performing NIPT before the FTAS, compared to 39.0% of PCPs. Implementing this sequence, where it is logistically feasible, may reduce unnecessary referrals [2] and improve cost efficiency.

### 4.2 Strengths and Limitations

The strengths of this study include a large sample size of both pregnant women and healthcare professionals. Another strength is the recruitment of pregnant women by the national prenatal registration database (Peridos), which enhanced the inclusion of participants from various regions in the Netherlands. Furthermore, since the FTAS has been available in the Netherlands in a study context since September 2021, healthcare professionals have gained experience with referrals following first‐trimester screening ultrasounds, allowing them to provide more informed opinions on their preferences.

Limitations of this study include the complexity of the DCE, as approximately one‐third of respondents (strongly) agreed that the questionnaire was difficult to complete, which may limit reliability. It remains unclear whether this complexity refers to challenges in understanding the DCE itself or in making choices between attributes. Participation was anonymous, limiting the ability to independently verify unique responses; however, all pregnant women were invited via personalized email. Additionally, all women opted for FTAS; most participants were Dutch and highly educated, potentially restricting generalizability to more diverse populations. Future studies should therefore aim to recruit a more diverse sample to determine whether preferences vary across educational and sociodemographic groups. Given the complexity of these choices within a DCE, more personalized approaches, such as qualitative interviews, could be employed to gain deeper insight into participant preferences.

## 5 Conclusions

Our findings indicate that pregnant women and healthcare professionals support the availability of the FTAS. Optimization should focus on maximizing sensitivity and minimizing false positive rates, whereas gestational age at diagnosis and the range of anomalies detected are considered less important in influencing preferences. These insights can guide policy and implementation decisions for the implementation of the FTAS within prenatal screening programs.

## Funding

The IMITAS study is supported by a grant from The Netherlands Organization for Health Research and Development (ZonMw, No. 543010001).

## Ethics Statement

Approval for the study was granted by the Dutch Ministry of Health, Welfare, and Sport (license 3219987‐1012059‐PG), based on positive advice by the Health Council of the Netherlands (2021/30). The present study was a predefined component of the ‘Implementation of First Trimester Anomaly Scan’ (IMITAS) study and was included in the original grant proposal and ethical approval.

## Consent

Online informed consent was obtained from all participants.

## Conflicts of Interest

The authors declare no conflicts of interest.

## Supporting information


Supporting Information S1

## Data Availability

The data that support the findings of this study are available from the corresponding author upon reasonable request.
