# Supplementary material for: Preferences of Pregnant Women and Healthcare Professionals on First‐Trimester Ultrasound Screening for Fetal Anomalies: A Discrete Choice Experiment
Source: Prenat Diagn. 2026 Mar 3;46(4):523–32. doi: 10.1002/pd.70115 (PMC13070217; doi:10.1002/pd.70115)

**Supplementary material**

**Supplemental S1:** Questionnaire

**Pregnant women**

| **Demographic information questions** | | |
| --- | --- | --- |
| Did you opt for an first-trimester anomaly scan after prenatal counseling? | 🞎 Yes  🞎 No | If no, exclusion |
| Have you already had the first-trimester anomaly scan during this pregnancy? | 🞎 Yes  🞎 No |  |
| What is your due date? | [date] |  |
| Is this your first pregnancy? | 🞎 Yes  🞎 No |  |
| How many pregnancies have you had, including this one? | [number] | If it is not the first pregnancy |
| How many living children do you have? | [number] | If it is not the first pregnancy |
| Did you have an first-trimester anomaly scan in a previous pregnancy? | 🞎 Yes  🞎 No | If it is not the first pregnancy |
| Have you ever been referred for a detailed diagnostic scan at the hospital after a first-trimester anomaly scan because of a suspected fetal anomaly? | 🞎 Yes  🞎 No | If someone had a first-trimester anomaly scan in a previous pregnancy |
| You have ever had an abnormal first-trimester anomaly scan. Which situation has ever applied to you?  If you have been referred multiple times after an abnormal first-trimester anomaly scan, you can select multiple options. | 🞎 At the first-trimester anomaly scan there was a suspected fetal anomaly, but at the first detailed diagnostic scan in the hospital, there was no longer a suspicion.  🞎 At the first-trimester anomaly scan and the first detailed diagnostic scan in the hospital, there was a suspected fetal anomaly, but when I was 24 weeks pregnant, no fetal anomalies were found anymore.  🞎 At the first-trimester anomaly scan, there was a suspected fetal anomaly and this was still the case when I was about 24 weeks pregnant.  🞎 At the first-trimester anomaly scan, there was a suspected fetal anomaly. I terminated the pregnancy or my child experienced intrauterine fetal demise.  🞎 Other | If someone had an abnormal first-trimester anomaly scan in a previous pregnancy |
| What kind of anomaly (or anomalies) was/were present? | [free-text] | If someone had a confirmed abnormal first-trimester anomaly scan in a previous pregnancy |
| What is your age? | [number] |  |
| In which province do you live? | [list of provinces] |  |
| What is your highest completed education? | 🞎 None  🞎 Primary school  🞎 LBO, MAVO (Lower vocational and general secondary education)  🞎 VMBO (Vocational education)  🞎 HAVO, VWO (General secondary education)  🞎 MBO (Intermediate vocational education)  🞎 HBO (Higher vocational education)  🞎 University  🞎 Prefer not to answer  🞎 Other |  |
| What is your ethnic or cultural background, as you identify it? | 🞎 Dutch  🞎 Western (e.g., European, North American, Australian)  🞎 Non-Western (e.g., Moroccan, Turkish, Surinamese, African, Asian)  🞎 Prefer not to say  🞎 Other |  |
| Do you have a religious belief? | 🞎 Yes  🞎 No  🞎 Prefer not to answer  🞎 Other |  |
| **Attitude questionnaire** | | |
| I believe the first-trimester anomaly scan should be available to pregnant women. | 🞎 Strongly disagree  🞎 Disagree  🞎 Neutral  🞎 Agree  🞎 Strongly agree |  |
| Could you briefly explain your answer to the previous question? | [free-text] |  |
| I found the questionnaire difficult to complete. | 🞎 Strongly disagree  🞎 Disagree  🞎 Neutral  🞎 Agree  🞎 Strongly agree |  |
| What did you think of the length of the questionnaire? | 🞎 Too short  🞎 Neither short nor long  🞎 Too long |  |
| If you have any additional comments, please write them below. | [free-text] |  |

**Healthcare professionals**

| **Demographic information questions** | | |
| --- | --- | --- |
| What is your profession? | 🞎 Prenatal counselor  🞎 Sonographer  🞎 Midwife  🞎 Maternal-Fetal Medicine Physician  🞎 OB/GYN  🞎 Pediatric specialist  🞎 Clinical geneticist | *Multiple answers possible* |
| Which pediatric subspecialty do you practice? | 🞎 Pediatric Cardiologist  🞎 Pediatric Urologist  🞎 Pediatric Nephrologist  🞎 Pediatric Neurologist  🞎 Pediatric Surgeon  🞎 Other | For pediatric specialists |
| In which setting do you work? | 🞎 Primary care  🞎 Secondary care  🞎 Tertiary care |  |
| How long have you been working in your field? | 🞎 Less than 1 year  🞎 1–2 years  🞎 3–5 years  🞎 6–10 years  🞎 11–15 years  🞎 More than 15 years |  |
| In which province do you work? | [list of provinces] |  |
| Do you perform ultrasounds yourself? | 🞎 Yes  🞎 No |  |
| Please indicate which types of ultrasounds you perform. | [list of different types of ultrasounds during pregnancy] | If applicable |
| **Attitude questionnaire** | | |
| I believe the first-trimester anomaly scan should be available to pregnant women. | 🞎 Strongly disagree  🞎 Disagree  🞎 Neutral  🞎 Agree  🞎 Strongly agree |  |
| The first-trimester anomaly scan should be permanently implemented into prenatal care. | 🞎 Yes, exactly as it is currently offered  🞎 Yes, but with modifications to the current offering  🞎 No, it should not be implemented  🞎 I don’t know |  |
| Could you briefly explain your answer to the previous question? | [free-text] |  |
| What is your opinion on the timing of the NIPT in relation to the first-trimester anomaly scan? | 🞎 The NIPT should take place before the first-trimester anomaly scan  🞎 The first-trimester anomaly scan should take place before the NIPT  🞎 Pregnant women should decide which test they undergo first  🞎 I don’t know  🞎 Other |  |
| If the NIPT is performed, should its result be awaited before performing the first-trimester anomaly scan? | 🞎 Yes  🞎 No  🞎 I don’t know  🞎 Other |  |
| I found the questionnaire difficult to complete. | 🞎 Strongly disagree  🞎 Disagree  🞎 Neutral  🞎 Agree  🞎 Strongly agree |  |
| What did you think of the length of the questionnaire? | 🞎 Too short  🞎 Neither short nor long  🞎 Too long |  |
| If you have any additional comments, please write them below. | [free-text] |  |

**Supplemental S2:** Example of a choice task


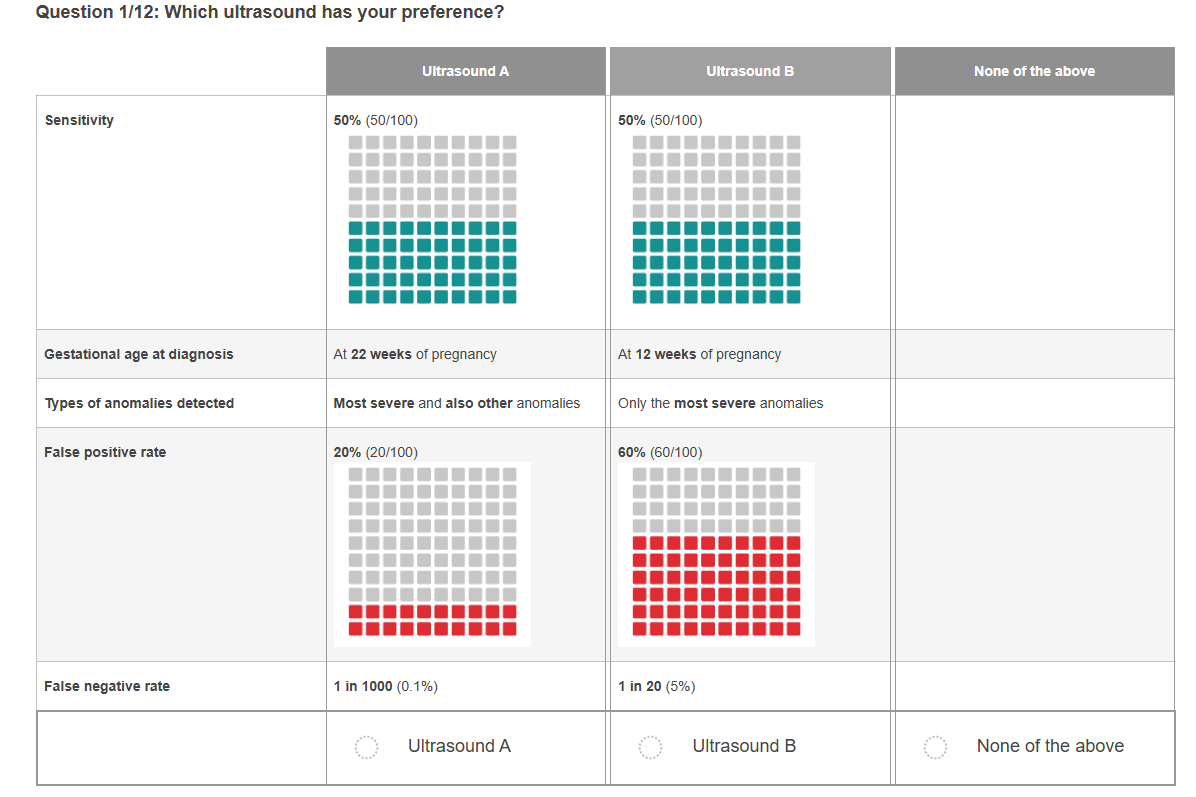

Supplement: Supplementary file 1 — Supporting Information S1 [file PD-46-523-s001.docx]
